# Molecular Biomarkers Related to Oral Carcinoma: Clinical Trial Outcome Evaluation in a Literature Review

**DOI:** 10.1155/2019/8040361

**Published:** 2019-03-25

**Authors:** Gabriele Cervino, Luca Fiorillo, Alan Scott Herford, Umberto Romeo, Alberto Bianchi, Salvatore Crimi, Cesare D'Amico, Rosa De Stefano, Giuseppe Troiano, Rossella Santoro, Luigi Laino, Gregorio Laino, Marco Cicciù

**Affiliations:** ^1^Department of Biomedical and Dental Sciences and Morphological and Functional Imaging, Messina University, Messina 98100, Italy; ^2^Department of Maxillofacial Surgery, Loma Linda University, Loma Linda, CA 92354, USA; ^3^Department of Oral and Maxillofacial Sciences, Paediatric Dentistry Unit, “Sapienza” University of Rome, Roma 00161, Italy; ^4^Department of Maxillofacial Surgery and Surgical Disciplines, University of Catania, Catania 98100, Italy; ^5^Departments of Clinical and Experimental Medicine, University of Foggia, 71121 Foggia, Italy; ^6^Multidisciplinary Department of Medical-Surgical and Odontostomatological Specialties, University of Campania “Luigi Vanvitelli”, 80121 Naples 80121, Italy

## Abstract

**Backgrounds:**

The objective of the present research was to systematically revise the international literature about the genetic biomarkers related to oral cancer (OC) evaluating the recent findings in clinical studies.

**Methods:**

A comprehensive review of the current literature was conducted according to the PRISMA guidelines by accessing the NCBI PubMed database. The authors conducted the search of articles in the English language published from 2008 to 2018. The present systematic review included only papers with significant results about correlation between wound healing, genetic alteration, and OC. Prognostic capacity of genetic markers was not evaluated in vivo.

**Results:**

The first analysis with filters recorded about 1884 published papers. Beyond reading and consideration of suitability, only 20 and then 8 papers, with case report exclusion, were recorded for the revision.

**Conclusion:**

All the researches recorded the proteomic and genetic alterations in OC human biopsy cells. The gene modification level in the different studies, compared with samples of healthy tissues, has always been statistically significant, but it is not possible to associate publications with each other because each job is based on the measurement of different biomarkers and gene targets. Further investigations should be required in order to state scientific evidence about a clear advantage of using these biomarkers for diagnostic purpose.

## 1. Introduction

Oral cancer (OC) is today considered one of the principal causes of deaths with an increasing distribution located in the developing countries. The difficulty in performing a quick diagnosis and prompt management seems to be the reason for this high mortality and morbidity. Recently, several investigation methods and modern instruments have been analyzed in order to help clinicians in doing noninvasive analysis and fast recognition of this kind of oral pathological lesions [1–9].

OC is a highly relevant problem of global public health, especially for dental surgeons. It is among the top 10 most frequent cancers, and though current research in the field discovered new therapies and treatment options, the survival still remains low representing a continuing challenge for the clinicians [7, 9–18].

A quick diagnosis is crucial in order to control a possible malignant transformation of oral premalignant diseases and for increasing the overall survival rate of the patients. Numerous techniques and methods like scraping the surface of the lesion analyzing the cytological characteristics of the oral premalignant lesions are essential for doing the right diagnosis. It is hard to state but clinicians should be able to recognize the features of the oral lesions just by doing a simple view and without touching the lesions avoiding possible modifications in the cells of the tissue [2–10, 16–24].

Nowadays, though the current standard of performing diagnosis in oral pathology is related to incisional biopsy with histology, this method is painful for patients and involves a delay in the diagnosis, although histology is fully done. A new technique for doing noninvasive analysis of a soft tissue lesion is the autofluorescence. It can be used as a helpful method useful to find oral precursor malignant lesions and the correct location for taking biopsies within the altered mucosa. However, the main limitation of this procedure is related to the possibility of frequently occurring false-positive results [1, 3, 18–20].

A novel issue in the OC diagnosis is connected to the molecular biology investigations. This procedure is able to highlight any modification at a molecular stage much before using a microscope and much before clinical changes happen.

Moreover, their molecular features can also classify oral lesions. So it is possible to predict malignant potential of oral lesions decreasing the incidence and to improve early diagnosis and treatment of OC [13, 21–29].

The progress into the understanding of human genome and the numerous possibilities of genetic and molecular researches can be used as diagnostic and prognostic tools for performing quick diagnosis and management of oral lesion by doing molecular investigation.

Molecular detection instruments can be classified into nucleic acid-based and protein-based markers. Nucleic acid-based modifications happen due to preceding epigenetic processes or existing genetic mutations, amplifications, and polymorphisms. These mechanisms lead to aberrant expressions of genes [30–36]. Unlike nucleic acid-based techniques, protein-based early detection tools detect posttranscriptional and posttranslational changes that may take place as a result of carcinogenesis. The reason of investigating the oral biomarkers available in the clinical study is related to the possibility of evaluating the soft tissue healing phases. In oral pathology, the wound healing physiological steps involve a complex interplay of cells, mediators, growth factors, and cytokines. The cascade of this inflammatory process starts with clotting and recruitment of inflammatory cells, and then, it proceeds to a highly proliferative state. At this time, fibroblasts are involved in the collagen matrix synthesis and remodelling. The keratinocytes spread across the wound to form a new epithelial layer, and angiogenesis occurs, regulating the tissue healing. A close correlation between specific OC biomarkers and wound healing should be significant in the whole health recovering inflammatory processes [1, 4, 7, 19, 36].

In this article, the authors will discuss genetic and molecular pathways as possible genesis of oral carcinoma. Clinical reports related to the soft tissue healing will be selected in order to determine useful prognostic and diagnostic factors for OC.

Moreover, the objective of the present revision is to overview the recent literature clinical trials based on diagnostic and prognostic possibilities of genetic and proteomic biomarkers of oral cancer.

## 2. Materials and Methods

### 2.1. Application Protocol and Website Recording Data

The inclusion parameters for the current research was collected in a protocol and then submitted in advance and documented in the CRD York website PROSPERO, an international prospective register of systematic reviews: application ID number: CRD 86658 (registration in progress).

The data of this systematic investigation observed the Preferred Reporting Items for Systematic Review accordingly with the PRISMA statement [37, 38].

### 2.2. Outcome Questions

The following next two questions were sentenced and structured according to the PICO study design:

- Are there some molecular biomarkers for oral carcinoma wound healing process?
- What is the diagnosis method for oral carcinoma, and what biomarkers are they using on clinical trials?

### 2.3. Searches

**Figure 1:**
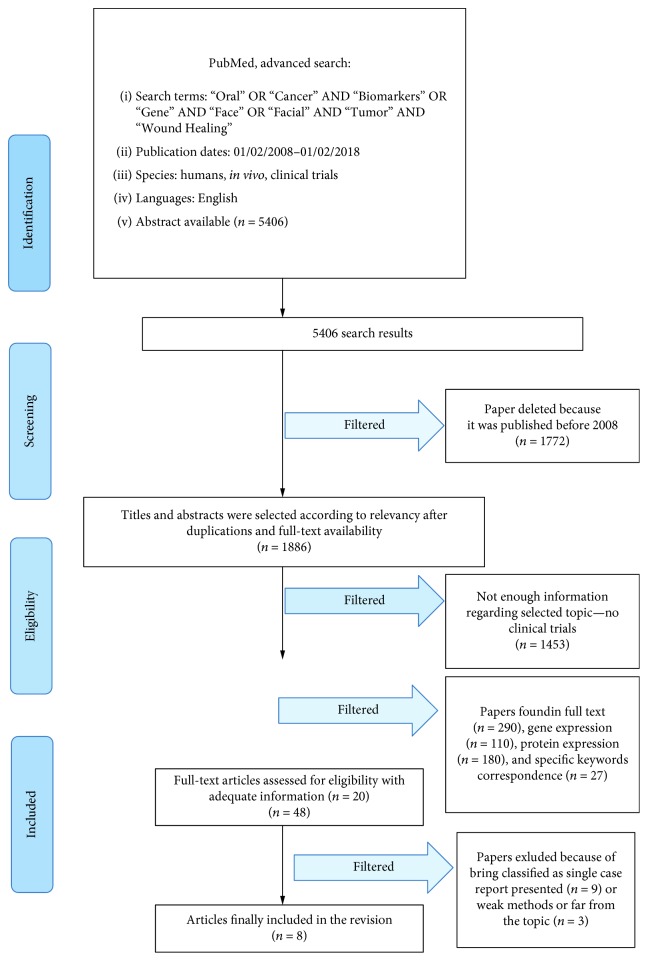
Prisma flow diagram.

The PubMed-Medline resource database was explored through advanced searches. The keywords and search inquiries used during the first selection stage were as follows: “oral cancer biomarker”, “oral cancer gene”, and “soft tissue wound healing”. Additional manually selected articles were included following the eligibility criteria.  Figure 1 represents the flow diagram of the selected studies according to guidelines and following the criteria for the investigated papers choice.

### 2.4. Data Recorded from the Selected Manuscripts

The Medical Subject Headings (MeSH) was applied for finding the keywords used in the present revision. The selected keywords: “oral” OR “facial” AND “cancer” OR “tumor” AND “biomarkers” AND “gene” AND “clinical” AND “wound healing”, were recorded for collecting the data.

### 2.5. Selections of the Papers

Four independent reviewers of different Italian Universities (Messina, Foggia, Catania, and Naples) singularly investigated the obtained full-text papers in order to select inclusion and exclusion criteria as follows. Reviewers compared decisions and resolved differences through discussion and consulting a third party when consensus could not be reached. For the stage of reviewing of full-text articles, a complete independent dual review was undertaken.

The manuscripts selected in the present revision highlighted the clinical researches on humans published in the English language. Letters, editorials, case reports, animal studies, and PhD thesis were excluded.

### 2.6. Research Classifications

The method of classification included all human prospective and retrospective clinical studies, split mouth cohort studies, case-control papers, and case series manuscripts, published between December 2008 and January 2018, on biomarkers for oral cancer and wound healing.

### 2.7. Statement of the Problem

The sentence case of “oral cancer biomarkers clinical trials wound healing” was searched over each selected papers.

### 2.8. Exclusion and Inclusion Criteria

The applied inclusion criteria for the studies were created as follows:

- English language
- Clinical human studies of oral cancer and molecular biomarkers
- Last ten-year data of publishing

The following types of articles were excluded as follows:

- In vivo/in vitro studies
- Studies of testing medication and/or new treatment methodologies
- Studies of cancer in locations other than mentioned
- Studies not relevant to our selected diagnostic methods
- Animal studies
- Literature review articles published prior to February 1st, 2008
- No access to the title and abstract in the English language

### 2.9. Strategy for Collecting Data

Following the initial literature search, all the article titles were screened in order to eliminate irrelevant publications, review articles, case reports, and animal studies. Next, studies were excluded based on data obtained from screening the abstracts. The final stage of screening involved reading the full texts confirming each study's eligibility based on the inclusion and exclusion criteria.

### 2.10. Data Extraction from the Collected Papers

The data and the results of the full-text manuscript screened were compared. The conclusions were used for assembling the data, according to the aims and themes of the present revision, as listed onwards.

The following key criteria were used as guidelines for agglomerating the data and then structured following the schemes:

- “Author (year)”—revealed the first author and the year of publication
- “Type of study”—indicated the method of the research
- “Sample origin”—describes the number of particular investigated samples in the study and its origin (e.g., BS: blood sample; SS: saliva sample; and TT: tumor tissue)
- “Follow-up”—yes/no described the duration of the observed outcomes
- “Result”—indicates the parameters that were coherent with alterations of particular biomarkers in prognostic studies

### 2.11. Risk of Bias Assessment

The grade of bias risk was independently considered and in duplicate by the two independent reviewers at the moment of data extraction process.

The quality of all included studies was assessed during the data extraction process. The quality appraisal involved evaluating the methodological elements that might influence the outcomes of each study. According to Moher et al. and Higgins et al., this revision followed the Cochrane Collaboration's two-part tool for assessing risk of bias and PRISMA statement [37, 38].

Risk of bias (e.g., absence of information or selective reports on variables of interest) was assessed on a study level. The risks were indicated as lack of precise information of interest related to the keywords selected.

This method applied by the four reviewers was valuable for giving to each study a level of bias. Then, the selected papers were classified with low, moderate, high, and unclear risk.

## 3. Results

### 3.1. Manuscript Collection

Manuscript choice and analyzing data process followed the PRISMA flow diagram (Figure 1). The first electronic and hand search performed on PubMed-Medline and Dentistry and Oral Sciences Source resulted with a total of 5406 papers. 1772 papers were excluded because they were published prior to February 1st, 2008. Then, the other 1886 papers were not involved in the revision because they were not available in full text. Then, the other 1453 papers were not selected because they were not directly developed as clinical trials. At this point, 290 titles and abstracts were evaluated: then, the papers were classified into papers that revealed gene expression *n* = 110 and protein expression *n* = 180; 27 articles were selected as having significant data regarding “Oral Cancer Tumor Biomarkers Clinical Trials Wound Healing” topic. 20 articles were determined as full-text papers, 8 of which were incorporated in this work. Some researches were excluded because of being classified as a single case report presented (*n* = 9) or weak methods or far from the topic (*n* = 3).

### 3.2. Statistical Analysis

No meta-analyses could be performed due to the heterogeneity between the studies (different study designs, control groups, and observation periods).

### 3.3. Study Characteristics

After the manuscript selection, a new time for screening related to the kind of gene expression or protein expression has been performed:

- Gene expression (*n* = 110)
- Protein expression (*n* = 180)

The final clinical papers in full text selected were numbered as 8.

### 3.4. Possible Bias of the Selected Studies

The possible risk of bias was evaluated for each selected papers. The final number of the selected papers was limited to eight papers. The inclusion criteria were really restrictive and for this reason also, the risk of bias was low. Seven studies were considered as having a low risk of bias [39–45]; another one was classified as moderate risk [46].

Current analysis of the data extracted from studies written in English only could introduce a publication bias. About possible bias, some of the selected papers did not specify the inclusion criteria of the patient selection. Another key parameter that can be assumed as bias is related to the evaluation of the clinical condition for selecting the patient. Some studies referred “patients with oral preneoplastic lesions,” while another study wrote about “patients with neoplastic lesion” [39, 43]. The soft tissue healing after the surgical excision was not evaluated in all the selected studies. Moreover, data recorded from the eight studies pointed out the heterogeneity of the research methods, selections of the patients, and therapeutic options. One paper started the investigation not directly from the patient but from immortalized human OSCC-derived cell lines (HSC-2, HSC-3, HSC-4, Ca9-22, Sa3, HO-1-u-1, and KON) obtained from the Human Science Research Resources Bank (Osaka, Japan) or the RIKEN BRC (Ibaraki, Japan) through the National BioResource Project of the Ministry of Education, Culture, Sports, Science and Technology, and this is another bias [46].

Tables 1 and 2 resume the studies selected and their results related to the altered biomarkers and to the biomarker measurements.

### 3.5. Genetic Alterations in Oral Cancer and Wound Healing

The chosen clinical papers evaluated the alterations in some gene expressions able to influence a predisposition by the patient on developing oral cancer and consequently the possibility on having a better healing. In the selected clinical studies, the oral cancer soft tissue biopsies have been recorded and then, the genetic expression of these biopsies was evaluated, highlighting any possible alterations. Alterations in the EGFR gene copy number, or alterations in miR-7, miR-21, mRNA-KIFGA, OPN, DEPDC1B, EZH2, deltaNp63, and DNMT3B were significant for early evaluation and correlation with oral cancer. It is fundamental to underline how sometimes the quick presumptive diagnosis of preoral cancer lesion and the stage of diagnosis remain the fundamental steps on recording positive oral cancer diagnosis. In the final 8 studies, the degree of significance of these data was never higher than *p* < 0.05. In one paper, the correlation between the patient's degree of survival and the expression of miR-21 is also considered. If the miR-21 values are high, the patient's chances of survival are lower. In one study, the degree of dysplasia is evaluated based on the expression of the EZH2 gene. Another study illustrated the possibility of evaluating the predisposition to the formation of OC by evaluating deltaNp63 and EIC, also using the expression of podoplanin [39–46].

In oncology, the tumor markers or tumor indicators are classified as substances that can be found in the blood or less often in the ascitic fluid, which show a significant increase in their concentration in some types of neoplasia. A high level of a tumor marker may indicate the presence of cancer, although other causes of raising those values may exist. Some markers are specific to certain tumors while others increase in many neoplasms. Tumor markers can be produced directly from tumor cells or from normal cells. The tumor markers, on the other hand, are more useful when they are used to monitor a possible recurrence of cancer after the treatment (surgical or medical) of the primary tumor. Many proteins are known to regulate programmed cell death (or apoptosis), and members of the Bcl-2 family are the most important example. This group includes at least 15 different proteins both with antiapoptotic function (Bcl-2, Bcl-X) and proapoptotic (Bax, Bak), and it represents the balance between these two activities determining cell fate. Regarding their role in the forms of OSCC, an increase in the levels of Bcl-2 and Bcl-X expression was observed, both in dysplastic oral lesions and in oral cancer [47]. p53 is a tumor suppressor involved in several mechanisms including cell cycle progression, differentiation, DNA repair, and apoptotic process regulation. p53, also known as tumor protein 53 (TP53 gene), is a transcription factor that regulates cell cycle and covers tumor suppressor function. It intervenes in many antitumor mechanisms, activates the repair of damaged DNA (if the DNA is repairable), and can initiate apoptosis, inducing the transcription of Noxa, in case DNA damage is irreparable; if the DNA is repaired, p53 is degraded and there is a recovery of the cell cycle. Some pathogens can instead directly affect the p53 protein. An example is the human papillomavirus (HPV), which encodes a protein which binds p53 inactivating it. This, in synergy with the inactivation of another cell cycle regulator, the p105RB, allows repeated cell divisions that occur in the clinical form of the wart. The introduction of p53 into cells with protein deficiency has shown to cause a rapid death of cancer cells or a block of cell division. This phenomenon reflects the possibility on having good therapeutic prognosis. For this reason, it is one of the most widely studied oral cavity biomarkers. The gene encoding is mutated in the 50% of the tumor forms, particularly in 25-69% of OSCC cases [48]. A high expression of p53 was observed in 40-67% of cases of carcinoma of the head and neck, and this variability is related to problems inherent in the method. Some authors [49, 50] have observed a direct relationship between overexpression of p53 and a poor prognosis in terms of survival. In other works, on the contrary, a correlation between p53 overexpression and survival did not clearly emerge, while an important role of p53 in the carcinogenesis process was highlighted, as an early event of malignant transformation, and of the histological progression of the tumor [51, 52]. The expression of p53 above the basal layer is considered an early event of the oral carcinogenesis process. It is an indicator of the development of carcinoma, even before the definite morphological changes of the involved tissue. The inactivation of this protein or the alteration of the coding gene could therefore play an important role in the genesis of OC. This could certainly represent a parameter (biomarker) to be taken into consideration during the diagnostic or interceptive phase of the tumor. Inactivated p53 is not able to stop the reproduction of cells with damaged DNA. This could be a starting point for OC. The Rb (retinoblastoma) pathway also plays a key role in regulating cell cycle progression, and this activity can be inhibited by specific mutations. Although Rb mutations are rare in oral cancer, its loss of expression was seen in 66% of OSCC cases and in 64% of premalignant lesions [22]. Another possible marker of oral cancer is Survivin, an apoptotic process inhibitor, expressed in about 80% of the forms of squamous cell oral carcinoma and whose expression is related with an aggressive phenotype [53]. It has been shown that miRNAs can have specific expression profiles for developmental stages, tissues, and various pathologies. Studies on several forms of cancer, including oral cancer, have shown an altered expression of miRNA in tumor tissue compared to healthy tissue, suggesting the involvement of these molecules in carcinogenesis [54–56]. Human cells have a limited capacity for self-replication and, after numerous cell divisions, cease to grow and enter on senescence phase. Cells with carcinogenic characteristics need to be immortal in order to replicate infinitely and succeed in maintaining the length of their telomeres unaltered.

Since tumor growth is limited to 1-2 mm3 in the absence of adequate perfusion, solid tumors require substantial blood supply to be able to grow and metastasize [57]. The angiogenic phenomenon is the result of the opposing action of proangiogenic signals (vascular endothelial growth factor (VEGF), platelet-derived growth factor (PDGF), and interleukin 8 (IL-8)) and antiangiogenic signals (interferons and proteolytic fragments such as angiostatin and endostatin). Oral squamous cell cancer has an important local invasive capacity and a high predisposition to metastasize in the cervical lymph nodes. The invasive and metastatic phenomena are the result of a series of processes involving cell adhesion, cytoskeletal rearrangement, cell migration and degradation of the basement membrane, passage and survival in the bloodstream, and the ability to escape from this and colonize distant sites with the formation of new vessels.

### 3.6. Proteomic Changes of Oral Cancer

A total of eight clinical studies, in which samples were analyzed, described protein biomarkers and evaluated the wound healing of the site after the surgery. In biology, a biomarker is a molecule that identifies the presence of a tissue. The marker can be of any nature, but substantially it is a protein, or otherwise polypeptide, since it is the proteins that are translated by DNA. For this reason, a marker is such: it is a molecule that is produced mainly by that type of cell. If the marker is used as a disease index, it should only be produced in the presence of this disease. Few markers however meet these needs. The major problem is given by tumor cell markers: as cells, however, are not completely extraneous to the body, neoplastic cells do not translate for molecules that make their dosage accurate method. From a molecular point of investigation, studies involved evaluated the aberrant expressions of candidate protein biomarkers and their quantitative yield in specimens. The protein modification is related to the genetic or epigenetic alterations. In some cases, the marker can be represented by high-density lipoprotein components, HDLs, and HDL-cholesterol, [16, 41–48] or even by genetic alterations such as those found in some solid tumors [50–54]. Proteins are fundamental for physiological cell functioning and life. Aberrant genetic expressions of potential proteins alter cell division, proliferation, immune response, tissue growth, and finally metastasis [48–55]. As for other kind OC cancers, typical patterns of protein expression or individual proteins with specific features have been recorded and classified as oral cancer biomarkers in order to perform diagnosis and therapy.

## 4. Discussion

The purpose of this review was to systematically overview published studies restricted to “clinical trials” concerning genetic and proteomic biomarkers for detection and prognosis of OC and their relation to wound healing.

Luo et al. [39] evaluated the role of osteopontin (OPN) in chemosensitivity in locally advanced oral squamous cell carcinoma (OSCC) in humans. Authors considered 121 patients and validated the role of OPN in cell proliferation. The recombinant human OPN was executed to SAS cells (human tongue carcinoma cell line) to investigate if the increased OPN protein could influence a proliferative advantage to SAS cells. The presence of OPN is related to bone resorption, wound repair, immune function, and angiogenesis. However, it is particularly strongly associated with tumorigenesis also. The authors demonstrated that the proliferation percentage was significantly increased in matricellular OPN in a dose-dependent manner in SAS cells. This result demonstrates that one of the major roles of OPN is to promote growth of OSCC cells. Moreover, it was concluded how OPN-mediated cisplatin resistance contributes to a poorer clinical outcome and local wound healing in patients with locally advanced inoperable OSCC treated with cisplatin-based IC and CCRT.

Taoudi Benchekroun et al. [40] performed a study investigating oral premalignant lesions. The authors obtained data indicating that an increased *EGFR* gene copy number is common. Therefore, it is associated with OSCC development in patients with oral premalignant lesions (OPLs) expressing high EGFR, particularly OSCC developing at the site of a high-expression OPL; the authors also suggested that EGFR inhibitors might prevent oral cancer in patients with OPLs having an increased *EGFR* gene copy number. Moreover, the authors also demonstrated that an increased *EGFR* gene copy number in OPLs is a precursor to *EGFR* gene amplification in HNSCC (as is chromosome 7 increased copy number) and an important oncogenesis-driving effector in oral oncogenesis reducing the possibility of having healing at the surgical site and final good prognosis for the patient.

Jung et al. [41] identified deregulated miRNAs in oral cancer and further focus on specific miRNAs that were related to patient survival. Authors reported that miRNA expression profiling provided more precise information when oral squamous cell carcinomas were subcategorized on the basis of clinic pathological criteria. Data extracted from their research highlighted that the interpretation of miRNA expression patterns could be better resolved when one takes into consideration clinical pathological data of OSCC subtypes. Patient survival data demonstrated that the keratinization and the high miR-21 levels were significant factors of OC patient prognosis. Moreover, miR-7 and miR-21, two keratinization-associated miRNAs, could influence the modification of the tumor suppressor gene RECK in OC. Even if the 17 analyzed tumors clinically showed similar features, unique miRNA expression patterns were generated for specific subtypes of OSCCs. Finally, the recorded data underlined that different clinicopathological features and miRNA expression profiles could be used as specific signatures of individual subtypes of oral tumors with different final prognoses and healing possibilities.

Minakawa et al. [46] assumed that Kinesin family member 4 (KIF4A) is involved in oral squamous cell carcinomas (OSCCs) pathogenesis by the activation of the spindle assembly checkpoint (SAC). KIF4A is overexpressed frequently in OSCC, which suggests interference in the function of the spindle checkpoint proteins such as BUB1, MAD2, and CDC20. KIF4A expression was correlated with tumor size in KIF4A-positive cases, suggesting that SAC activation plays a significant role in cellular proliferation in OSCC. The authors concluded that KIF4A expression is likely to be a key regulator of carcinogenesis progression in OSCCs.

Su et al. [42] studied how the DEPDC1B (defined like guanine nucleotide exchange factor) induced both cell migration in a cultured embryonic fibroblast cell line. Moreover, it was recorded to favor anchorage-independent growth in oral cancer cells. It was demonstrated that DEPDC1B exerts a biological function by regulating Rac1. To determine whether DEPDC1B played a role in the induction of cell proliferation, contributing to faster wound healing, the authors evaluated the growth rate of cells expressing DEPDC1B and control cells founding no substantial difference between the growth rates of DEPDC1B-expressing cells and control cells.

However, the authors concluded that oral cancer samples overexpressed DEPDC1B proteins, compared with normal adjacent tissue, and so DEPDC1B plays a role in the development of oral cancer.

Cao et al. [43] investigated the role of the transcriptional repressor named Enhancer of Zeste Homolog 2 (EZH2) in oral carcinogenesis and its clinical implication as an OSCC risk predictor. The study revealed how at 5 years after diagnosis, the 80% of patients whose OLs expressed strong EZH2 developed OSCC. In Leuk-1 cells, EZH2 downregulation resulted in G1 arrest, decreased invasion capability, decreased anchorage independent growth, downregulation of cyclin D1, and upregulation of p15^INK4B^. The recorded data suggested that EZH2 seems to have a fundamental role in OL malignant transformation and may be a biomarker in predicting OSCC development in patients with OLs. Moreover, classifying the EZH2 expression in three stages as weak, moderate, and strong, the authors correlated this situation with better or not clinical healing, patient survival, and final prognosis. Quick diagnosis results are fundamental in order to approach the right therapy and for long survival.

Saintigny et al. [44] considered deltaNp63 as homolog of the p53 tumor suppressor and frequently amplified and overexpressed in squamous cell carcinomas, including head and neck squamous cell carcinoma. The authors were able to determine, in a relatively large population from whom OPL samples had been collected in a prospective longitudinal manner, how the level of overexpression of deltaNp63 alone or in combination with other molecular and morphologic features can be associated with a high risk to develop oral cancer. This investigation referred only oral cancers that developed in the same site as the OPL; 25% of the patients positive for podoplanin developed cancer, compared with 4% of the patients negative for podoplanin; 24% of the patients positive for deltaNp63 developed cancer, compared with 7% of the patients negative for deltaNp63; and 40% of the patients positive for all the biomarkers developed oral cancer, compared with 9% of the patients with no, one, or two positive biomarkers. The authors concluded that because the measurement of the three biomarkers can be done in routine pathology laboratories, it can be useful for evaluating soft tissue healing after OC removal and then patient survival.

Saintigny et al. [45] in a next investigation tried to determine the value of gene expression profiling in predicting oral cancer development. Gene expression profile was measured in 86 of 162 OPL patients who were enrolled in a clinical chemoprevention trial that used the incidence of oral cancer development as a prespecified endpoint. The results showed that gene expression profiles might improve the prediction of oral cancer risk in OPL patients. Moreover, the significant genes identified may serve as potential targets for oral cancer chemoprevention. Tumor progression from normal mucosa to dysplastic mucosa and eventually cancer is the result of a series of gene modifications affecting the normal functions of genes such as protooncogenes and tumor suppressors. Such alterations can be partly inherited but most are mutations that develop ex novo and accumulate in precancerous and cancerous tissue. These mutations can cause alterations in cell cycle regulation, differentiation, proliferation, DNA repair mechanisms, and cellular immunity. Chromosomal aberrations such as deletions, amplifications, and structural rearrangements are common in neoplasms and therefore also in head and neck cancer.

All those clinical studies evaluated an alteration of genomic proteins leading a tissue transformation directed to the OC formation. The possibility of quickly knowing those entire factors such as oral premalignant lesions (OPLs) may help in quick diagnosis and management.

Unfortunately, among the studies taken into consideration, few of those evaluate the same markers; thus, the risk of bias of this review study is classified as “high.” It is not possible to make a real report of the statistics of the different studies, which, however, despite the small number of patients have satisfactory statistical results. It is very interesting to consider the possibility that these biomarkers represent a factor to intervene early in the pathology so as to make complex reconstructions more rare [57]. Specifically in those cases, clinicians should avoid the use of complex rehabilitations placing dental implant fixtures increasing chronic inflammatory process of the jaws and exposing the patient to a risk [58]. In this way, the risk of psychological complications can be reduced and it can affect the patients' oral health and quality of life [59]. It is interesting to highlight anomalies in the crevicular fluid associated with the inflammatory state of the mucosa therefore with precancerous lesions, benign lesions, or OC [60, 61].

## 5. Conclusions

It is estimated that in the world, the annual cases of squamous cell head/neck neoplasia are more than 640,000 (with 350,000 deaths). After the success in HER2-positive metastatic breast cancer, lapatinib (a small oral molecule that is the result of GSK research) has also opened an important path in the treatment of head and neck cancer. These results tell us that the use of a dual tyrosine kinase inhibitor such as lapatinib may be clinically important not only in breast cancer but also probably in other tumors such as the head and neck, where EGFR is overexpressed. Surely, the possibility of identifying markers for a diagnosis of a primary oral cavity tumor or a relapse, especially if early, can save the life of numerous patients. The possibility of having a set of biomarkers that represent a certain risk for OC and above all the ease of sampling may constitute real screening for all patients at risk (genetic predisposition or family history) or exposed to environmental risks (alcohol, smoking, etc.). The present systematic review of clinical studies discovered genes and proteins associated with OC and strictly related with the wound healing, the prognosis, and patients' long-term survival. Due to high heterogeneity of the researches, it was not possible to perform meta-analysis for comparing the data of the selected papers. Due to poor materials and several parameters recorded, it is not possible to establish biomarkers specific for oral cancer. The diagnostic capabilities are also not sufficiently developed and used to allow the use of these markers. However, the highlighted papers demonstrated how the high, low, or moderate marker expression might influence the clinical status and the final prognosis of the patients. At this stage, it seems not possible to define standard genetic patterns of tumor cells.

## Figures and Tables

**Table 1:** Altered biomarkers in OC.

#	Year	Author	Subjects (*n*)	Sample origin^∗^	Gene marker^∗∗^	Result	*P* value
1	2010	Taoudi Benchekroun et al.	162	HB	EGFR (U)	An increased EGFR gene copy number increases the risk of OSCC	*P* = 0.062
2	2012	Jung et al.	17	TB	134 different miRNA (see image 1)	Keratinization and high miR-21 levels are important indicators of oral cancer patient prognosis	*P* < 0.05
3	2013	Minakawa et al.	106	TB	KIFGA (U)	Results showed that KIFGA is overexpressed in OC	*P* < 0.05
4	2015	Luo et al.	121	HB	OPN (osteopontin)	Tumor OPN plays an important role in tumor development particularly in tumor invasion and metastasis	*P* = 0.002
5	2014	Su et al.	7	HB	DEPDC1B (U)	DEPDC1B is highly expressed in oral cancer tissue, compared to adjacent tissue. The overexpression in cells promotes cell migration and induces cell invasion in cancer cell lines	/
6	2011	Cao et al.	76	TB	EZM2(D)	EZH2 expression is an independent predictor for OSCC. EZH2 may serve as a biomarker for oral cancer risk	*P* = 0.05
7	2009	Saintigny et al.	162	HB	deltaNp63 (U), EIC (U), podoplanin (U)	Hazard risk of OC with upregulated genes is augmented. Considering all three biomarkers, OC patient survival rate is strikingly higher compared with no, one, or two positive biomarkers	*P* < 0.0001
8	2011	Saintigny et al.	162	HB	Has-miR-101 (D), deltaNp63 (U), P63 (U), DNMT3B (U)	It demonstrated the value of gene expression profiles in predicting oral cancer development in OPL patients. The microRNA-based strategies might therefore be considered in future chemoprevention studies	/

^∗^Type of sample: HU: human biopsy; TB: tissue bank sample. ^∗∗^Type of altered gene regulation: D: downregulation, diminution; U: upregulation, augmentation.

**Table 2:** Biomarker measurement.

#	Year	Author	Subjects (*n*)	Sample origin^∗^	Gene marker^∗∗^	Sample preparation	Method
1	2010	Taoudi Benchekroun et al.	162	HB	EGFR (U)	Human OC biopsy formalin fixed and paraffin-embedded	FISH
2	2012	Jung et al.	17	TB	134 different miRNAs (see image 1)	Cell culture and transfection of oral cancer cells and normal cell biopsy	mirVana™, microarray gene expression, qRT-PCR
3	2013	Minakawa et al.	106	TB	KIFGA (U)	Immortalized human OSCC-derived cell lines obtained from the tissue bank. Human biopsy fixed in 20% buffered formaldehyde solution	qRT-PCR
4	2015	Luo et al.	121	HB	OPN (osteopontin)	Human OC biopsy formalin fixed and paraffin-embedded	Western blot
5	2014	Su et al.	7	HB	DEPDC1B (U)	Human biopsy	Immunoprecipitation, Northern blot, Western blot
6	2011	Cao et al.	76	TB	EZM2(D)	Human biopsy sample paraffin included and sectioned. Colored with H&E	Western blot
7	2009	Saintigny et al.	162	HB	deltaNp63 (U), EIC (U), podoplanin (U)	Human OC biopsy formalin fixed and paraffin-embedded	Cell membrane immunoreactivity, microscope
8	2011	Saintigny	162	HB	Has-miR-101 (D), deltaNp63 (U), P63 (U), DNMT3B (U)	Whole biopsy including both the epithelial cells and the underlying stroma	Microarray gene expression

^∗^Type of sample: HU: human biopsy; TB: tissue bank sample. ^∗∗^Type of altered gene regulation: D: downregulation, diminution; U: upregulation, augmentation.
